# Evaluation of therapeutic efficacy of ^211^At-labeled farletuzumab in an intraperitoneal mouse model of disseminated ovarian cancer

**DOI:** 10.1016/j.tranon.2020.100873

**Published:** 2020-09-25

**Authors:** Stig Palm, Tom Bäck, Emma Aneheim, Andreas Hallqvist, Ragnar Hultborn, Lars Jacobsson, Holger Jensen, Sture Lindegren, Per Albertsson

**Affiliations:** a Department of Radiation Physics, Institute of Clinical Sciences, Sahlgrenska Academy, University of Gothenburg, Gothenburg, Sweden; b Region Västra Götaland, Sahlgrenska University Hospital, Department of Oncology, Gothenburg, Sweden; c Department of Oncology, Institute of Clinical Sciences, Sahlgrenska Academy, University of Gothenburg, Gothenburg, Sweden; d Cyclotron and PET Unit, KF-3982, Rigshospitalet, Copenhagen, Denmark

## Abstract

**Introduction:**

Antibodies labeled with alpha-emitter astatine-211 have previously shown effective in intraperitoneal (i.p.) treatments of ovarian cancer. In the present work we explore the use of investigational farletuzumab, aimed at the folate receptor alpha. The aim was to evaluate the biodistribution and therapeutic effect of ^211^At-farletuzumab in in-vitro and in-vivo experiments and, using models for radiation dosimetry, to translate the findings to expected clinical result. The activity concentration used for therapy in mice (170 kBq/mL) was chosen to be in agreement with an activity concentration that is anticipated to be clinically relevant in patients (200 MBq/L).

**Methods:**

For biodistribution, using intravenous injections and mice carrying subcutaneous (s.c.) tumors, the animals were administered either ^211^At-farletuzumab (n = 16); or with a combination of ^125^I-farletuzumab and ^211^At-MX35 (n = 12). At 1, 3, 10 and 22 h, mice were euthanized and s.c.-tumors and organs weighted and measured for radioactivity. To evaluate therapeutic efficacy, mice were inoculated i.p. with 2 × 10^6^ NIH:OVCAR-3 cells. Twelve days later, the treatments were initiated by i.p.-administration. Specific treatment was given by ^211^At-labeled farletuzumab (group A; n = 22, 170 kBq/mL) which is specific for OVCAR-3 cells. Control treatments were given by either ^211^At-labeled rituximab which is unspecific for OVCAR-3 (group B; n = 22, 170 kBq/mL), non-radiolabeled farletuzumab (group C; n = 11) or PBS only (group D; n = 8).

**Results:**

The biodistribution of ^211^At-farletuzumab was similar to that with ^125^I as radiolabel, and also to that of ^211^At-labeled MX35 antibody. The tumor-free fraction (TFF) of the three control groups were all low (PBS 12%, unlabeled specific farletuzumab 9% and unspecific ^211^At-rituximab 14%). TFF following treatment with ^211^At-farletuzumab was 91%.

**Conclusion:**

The current investigation of intraperitoneal therapy with ^211^At-farletuzumab, delivered at clinically relevant ^211^At-mAb radioactivity concentrations and specific activities, showed a 6 to 10-fold increase (treated versus controls) in antitumor efficacy. This observation warrants further clinical testing.

## Introduction

In the quest for new and better treatments of disseminated cancers, targeted alpha-particle therapy (TAT) hold promise [1]. Use of a targeted monoclonal antibody, radiolabeled with the relatively short half-life alpha-emitter astatine-211 (*t*_½_ = 7.2 h), is particularly attractive for therapy of microtumors confined within a local compartment separated from the most radiosensitive organs. To explore this, we have focused on evaluating and optimizing adjuvant TAT of ovarian cancer, where any remaining microscopic tumors are typically located within the peritoneal cavity. We have previously translated promising in-vitro and pre-clinical evaluation of intraperitoneally administered ^211^At-MX35 [2,3] to a safety-profile seeking clinical phase I study [[4], [5], [6]].

The generated knowledge has allowed us to construct predictive models [7,8] that can assist in optimizing future patient therapies. One of the findings was that a high-affinity antibody, e.g. MX35, together with a relatively short half-life alpha emitter, e.g. ^211^At, is unlikely to penetrate substantially into microtumors, due to the so-called binding barrier. The result will be that the core of microtumors with diameters larger than ~200 μm will escape irradiation.

To partly overcome this shortcoming, we have herein evaluated farletuzumab (MORab003; Eisai Inc.) as a candidate vector for intraperitoneal TAT. Farletuzumab is an optimized anti-folate receptor alpha (FRA) antibody and is the humanized version of the murine LK26 [9]. It targets the human FRA which is overexpressed in 80–100% of epithelial ovarian cancers [[10], [11], [12], [13]]; is retained on metastatic foci and recurrent tumors [14]; and the receptor expression is associated with biologic aggressiveness and tumor phenotype [11].

Farletuzumab has relatively low binding affinity, 2 nM [15], which could serve to increase the penetration into microtumors, and therefore possibly eradicate microtumors larger than what is possible with a high-affinity antibody [8]. This is particularly attractive in an adjuvant clinical setting where microtumors up to ~1 mm might be present. Since FRA-expression is almost absent in normal tissues, farletuzumab is a promising vector for adjuvant intraperitoneal radioimmunotherapy.

The aim of the current study was to evaluate the biodistribution and therapeutic efficacy of ^211^At-farletuzumab in in-vitro and in-vivo experiments and, using models for radiation dosimetry, to translate the findings to expected results in the clinical setting. The experiments were therefore designed to mimic the clinical situation regarding mAb concentration and radiation doses.

## Methods

### Monoclonal antibodies and cells

The investigational antibody farletuzumab (MORab003, fully humanized IgG) is directed against human folate receptor alpha (FRA) which has elevated expression in approximately 90% of ovarian cancers [16]. Farletuzumab was produced and provided by Morphotek Inc. (Exton, PA, USA). The murine IgG mAb MX35, directed against the NaPi2b cell surface glycoprotein, was developed at the Memorial Sloan-Kettering Cancer Center (New York, NY, USA) and was produced from hybridoma cells obtained from the Ludwig Institute for Cancer Research (Zürich, Switzerland). Rituximab (chimeric anti-CD20, IgG) was obtained from the Swedish Pharmacy, Sahlgrenska University Hospital, and was used as unspecific control antibody.

NIH:OVCAR-3 cells were obtained from ATTC (Rockville, MD, USA). The cells were cultured in RPMI 1640 cell media without folic acid (Biowest) and supplemented with 10% fetal bovine serum, 1% l-glutamine and 1% penicillin and streptomycin. Cultures were adherent, fed twice a week, and split once a week. In all subsequent experiments, where relevant, RPMI 1640 cell media without folic acid was used.

### Radionuclides

Astatine-211 was produced at Copenhagen University Hospital, Denmark, via the ^209^Bi(α,2n)^211^At reaction in a Scanditronix MC32 cyclotron. After courier transport to Gothenburg (Sweden), ^211^At was isolated using a dry distillation procedure and recovered in chloroform, as previously described [17,18]. Iodine-125 was purchased from Perkin Elmer Sverige AB (Sweden).

### Antibody conjugation and radiolabeling

The antibodies were conjugated with N-succinimidyl 3-(trimethylstannyl)benzoate (m-MeATE) and radiolabeled as previously described [19]. Briefly, the ^211^At chloroform solution was evaporated to form a dry residue. For labeling the ^211^At, dry residue was activated with N-iodosuccinimide in methanol/1% acetic acid. The m-MeATE-immunoconjugate was acidified to a pH of ~5.5 using citric acid and then added to the activated ^211^At. An astatine activity of 50–200 MBq was used depending on the desired specific activity. The activated astatine was contacted with the immunoconjugate for 1 min on a vortex shaker. The reaction was quenched with sodium ascorbate and the astatine-labeled immunoconjugate was then purified into PBS by size exclusion chromatography (NAP-5, GE HealthCare). Radiolabeling with ^125^I was done as previously described [20].

### Mice

Six-weeks-old female BALB/c nude mice were purchased from Janvier Labs (France) and housed at the local animal research facility and kept according to the Animal Welfare Act (2018:1192) under the Swedish Board of Agriculture. Mice were allowed one week of acclimatization before tumor-cell inoculation. Water and food was provided ad lib. This study was approved by the local animal experiments committee (Gothenburg, Sweden, permit 89-2014).

### Binding kinetics

#### Mathematical model

A model for antibody binding to cells as well as penetration in microtumors has previously been presented for antibody concentrations used in human intraperitoneal TAT [8]. In order to use this model also for farletuzumab kinetics, parameters values for *k*_*on*_, *k*_*off*_, and the number of antigens available per cell needed to be established. Several cell experiments (outlined below) were designed for this purpose.

#### Cell-binding kinetics

The cell-binding kinetics was determined using the same antibody concentration as that which is proposed [8] for clinical intraperitoneal ^211^At-farletuzumab therapy, i.e. and antibody concentration of 0.4 μg/mL. OVCAR-3 cells, 2 × 10^6^ cells in 2 mL, were incubated at 37 °C with ^125^I-farletuzumab for 1, 2, 3, 4, 5, or 24 h. The cell suspension was then transferred to a new tube and centrifuged 4 min. Cells were washed twice in medium and the cell pellet and the final supernatant were separately measured for radioactivity.

#### Off-rate constant (*k*_*off*_)

Separate experiments (n = 15) were designed to establish the release component *k*_*off*_. In these, 2.6 × 10^7^ OVCAR-3 cells in 13 mL supplemented RPMI-1640 medium was added to a 50-mL tube and incubated with ^125^I-farletuzumab (0.4 μg/mL) for 1–5 h. Cells were then washed by first adding 35 mL medium and then centrifuged for 4 min followed by removal of the supernatant. Another 13 mL medium was added to the cell pellet to form a cell suspension that was transferred to six tubes (2 mL, holding 2 × 10^6^ cells) for *k*_*off*_ determination at six time-points, 1–24 h, when 2 or 40 mL were added to the respective tube. Cells were washed and the cell pellet and the supernatant were each measured for radioactivity.

#### Saturation experiments

One set of experiments (n = 5) were designed to evaluate the number of relevant antigens available per cell. Antibody concentrations ranged from what is anticipated for clinical used, i.e.0.4 μg/mL up to 40 μg/mL, i.e. 100 fold higher. Cell binding were first evaluated at 1, 2, 3, 4, 5, and 24 h. Since saturation was reached within 1 h, repeat experiments were evaluated only at 2 h.

#### SPR studies

Surface Plasmon Resonance (SPR) investigations of both unconjugated and m-MeATE-conjugated farletuzumab were performed using a Biacore 2000 system. The antibody/antibody conjugate was immobilized in two channels on a Protein A chip (GE Healthcare, 50 μg/ml, 30 μl/min up to circa 5000 RU, resonance units) and the Folate alpha antigen was dissolved in the running buffer (10 mM Hepes, 150 mM NaCl, 3 mM EDTA, 0,005% v/v P20, pH 7.4 (NaOH)). Between each folate alpha concentration (200 μl per concentration, run in the following order to avoid systematic error 0, 10, 1, 5, 10, 10, 30, 0, 80 nM) the chip was completely regenerated (using 10 mM Glycine-HCl, pH 2.3) and the protein immobilized again. This was repeated with low variability of the response for the same concentration. Binding kinetics data were calculated using a 1:1 Langmuir isotherm binding model.

#### IRF studies

The immunoreactive fraction of ^211^At-labeled farletuzumab was determined according to Lindmo et al. [21] using OVCAR-3 cells grown in folate-free media. Cells (5 × 10^6^ cells/mL) were serially diluted (1:2, six times) using PBS/BSA (0.1% BSA) in duplicates for each experiment. A constant amount (0.5–5 ng) of radiolabeled immunoconjugate was added to every sample. The association was allowed to proceed for 180 min at room temperature under gentle agitation. The cells were then centrifuged (3500 rpm, 5 min) to form a pellet and the supernatant removed. PBS was then added whereby the cells were re-suspended and subsequently centrifuged again before removal of the supernatant and measuring for radioactivity. The total radioactivity added to each sample (determined through reference samples) was compared to the activity of the cell samples to determine the bound immunoconjugate fraction.

### Biodistribution

Mice (n = 28) were subcutaneously inoculated at one or two sites, each with 1 × 10^7^ NIH:OVCAR-3 cells in 0.1 mL. Four weeks thereafter, the longest axes of the tumors were 5–7 mm, and mice were i.v. injected (0.1 mL in the tail vein) with radiolabeled antibodies.

One group was administered with 450 kBq ^211^At-farletuzumab (n = 16) and another group with a combination of 150 kBq ^125^I-farletuzumab and 450 kBq ^211^At-MX35 (n = 12). The MX35 was included as positive control of specific tumor uptake known from previous studies. At 1, 3, 10 and 22 h, mice were euthanized and tumors and organs weighed and measured for radioactivity in a gamma-well counter (3¨ NaI-detector; Wizard 1480; PerkinElmer Life Sciences). The measurements were corrected for physical decay and dead-time errors. Activity of ^211^At was determined by measuring the 77–93 keV characteristic X-rays and correcting for their abundance (43 photons per 100 ^211^At disintegrations). Since the samples contained both ^125^I (*t*_½_ = 59.4 days) and ^211^At (*t*_½_ = 7.21 h) they were all measured at two times in two separate energy windows. The first measurement was made directly after dissection of the organs and the second after three days when all ^211^At had decayed. This way the ^125^I activity could be measured without spill-over errors from ^211^At to the ^125^I-window, while for ^211^At the correct activity could be derived by subtracting the spill-over counts from ^125^I to the ^211^At-window.

### Therapeutic efficacy study on microscopic tumors

Therapeutic efficacy was investigated on sixty-three mice sorted into four groups. All mice were inoculated i.p. with 2 × 10^6^ NIH:OVCAR-3 cells in a single-cell suspension (0.2 mL PBS). Active or control (sham) treatments, all in 0.7 mL, were delivered i.p. twelve days after cell inoculation. The experiment was designed so that by the chosen injected activity, the microtumors in mice would receive roughly the same absorbed dose as microtumors in patients (following an administration of ^211^At-farletuzumab at 200 kBq/mL, which is anticipated for clinical treatments). The rate of antibody escape from the abdominal cavity in mice [22] is higher than in patients [7]. To adjust for this, a concentration roughly five times higher than what is proposed for clinically use was delivered to the mice, i.e. 1000 kBq/mL.

The experimental group (A; n = 22) received 700 kBq ^211^At-farletuzumab together with trace amounts (10 kBq) of ^125^I-rituximab. One control group (B; n = 22) received 0.7 MBq unspecific ^211^At-Rituximab. Another control group (C; n = 11) received unlabeled farletuzumab; and one group (D; n = 8) received PBS only.

The mice were inspected for well-being every second day and weighed every 7 to 10 days. At signs of deteriorating health, animals were euthanized and the abdominal cavity opened to investigate any presence of ascites and/or macroscopic tumors. Remaining mice were euthanized and evaluated 5 months (154 days) after therapy. To investigate occurrence of microscopic tumors in mice without established ascites or macroscopic tumor, tissues from the abdominal wall, mesentery, and spleen were taken for paraffin sectioning and hematoxylin and eosin (H&E) staining. Additional samples were taken from suspected lesions at other locations. Between 10 and 30 sections at 100 μm distance were processed for microscopy. Both the animal dissection and the histological analysis were blinded, i.e., performed without any knowledge of the treatment received.

Fisher Exact Test (Graphpad PRISM) was used for determining the significance of difference in TFF between mice treated with ^211^At-farletuzumab and each of the three control groups.

### Alpha-camera imaging and uptake estimates

In a separate experiment, imaging and quantification of ^211^At-labeled antibodies' uptake in intraperitoneal microtumors was conducted using the Alpha Camera system [23]. Two weeks following intraperitoneal cell inoculation (as specified for the therapeutic efficacy study; above), the mice were i.p. injected with ^211^At-farletuzumab or ^211^At-MX35. Within each group, half the mice were administered an activity concentration of 170 kBq/mL and the other half 5.7 MBq/mL. Alpha imaging was conducted at 4 h and 9 h after injection, as previously described [24] Since previous studies have shown that microtumors are typically located on the dorsal side of the spleen [24], the spleens were removed and snap-frozen in cryoprotective gel using liquid nitrogen. Sections consecutive to those used for alpha images were taken for H&E-staining and used for identification of areas with microtumors on the peritoneal lining of the spleen. Following co-registration with the alpha-sections, these areas were used to delineate regions-of-interest (ROIs) in the alpha images. For each tumor lesion, the activity uptake was quantified from the alpha-imaged section and the number of tumor cells in the micro-tumor was estimated from the H&E-section.

### Radiation dosimetry

An in-house-developed model of the biodistribution of i.p. infused antibodies in humans [7] was modified for the kinetics expected in mice. In addition, the binding kinetics data derived from the in-vitro experiments described in the current work was used for estimating the time-varying amounts of radiolabeled antibodies on single cells and cell clusters of varying sizes (expected to be present i.p. at the time for therapy [3]). Another in-house-developed Monte Carlo program [25] then allowed the calculation of mean absorbed dose to microscopic tumors.

Mean absorbed dose to organs and macrotumors was calculated using the biodistribution data, using methods described previously [26].

## Results

### Antibody radiolabeling

Astatine-211 labeling of m-MeATE-conjugates resulted in 65 (±7) % labeling yield and 96 (±3) % purity, both for farletuzumab and MX35. The overall radiochemical yield was 63 (±7) %. Specific activities up to 2.3 GBq/mg, corresponding to 1 in 45 antibodies being radiolabeled, were achieved. The iodine-125 labeling of the MeATE reagent and subsequent conjugation to farletuzumab resulted in an overall radiochemical yield of 38% (±5) and the radiochemical purity was always >95%. The specific activity of the iodinated antibodies was adjusted to match the specific activity of the astatinated farletuzumab.

### Binding kinetics

**Fig. 1:**
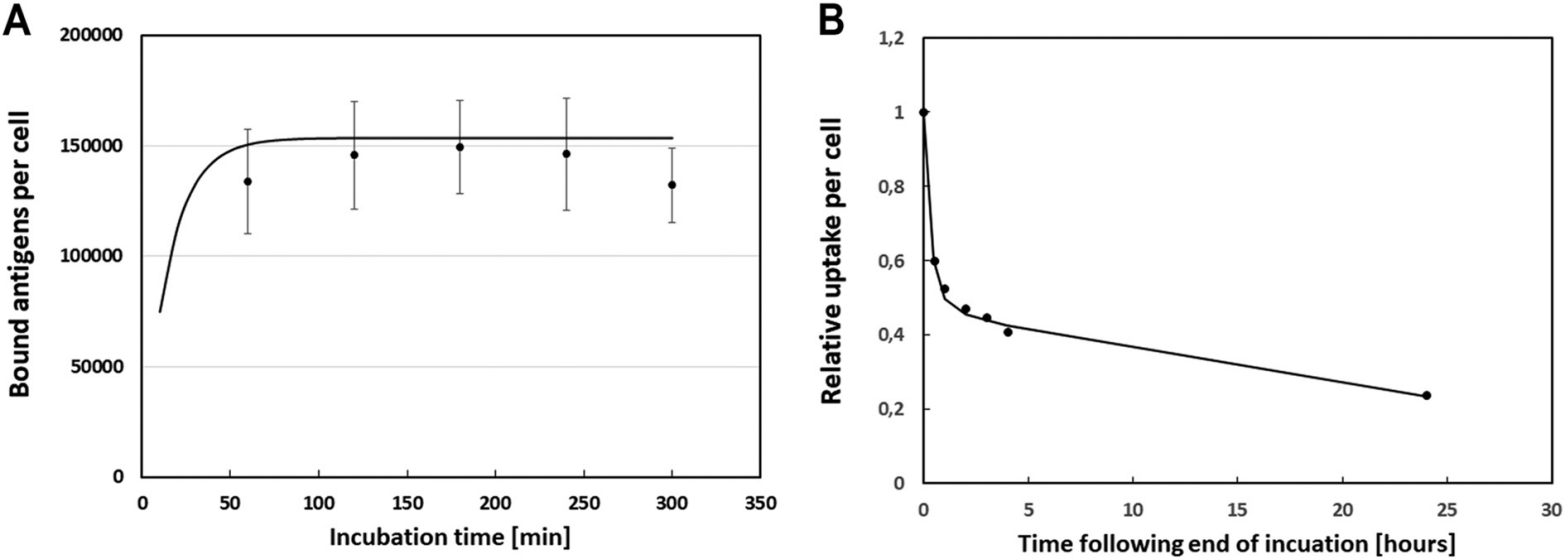
^125^I-farletuzumab binding to OVCAR-3 cells for various incubation times followed by washings (A). Panel B shows the relative remaining amount of ^125^I-farletuzumab per cell at various times after incubation has ended. Solid lines show model results when applying equal amounts of two sets of binding parameters (one set using k_on_ = 5.06 × 10^5^ M^−1^ s^−1^ and k_off_ = 5.61 × 10^−3^ s^−1^ and the other set using k_on_ = 2.25 × 10^5^ M^−1^ s^−1^ and k_off_ = 5.02 × 10^−4^ s^−1^) for the mAb binding kinetics to 600,000 antigens per cell, and ignoring possible internalization.

For antibody concentrations anticipated for clinical intraperitoneal TAT, i.e. 0.4 μg/mL, the uptake of ^125^I-farletuzumab on OVCAR-3 cells reached equilibrium within 1 h (Fig. 1A). The measured number of bound mAbs per cell was 150,000 after a wash. Comparisons head-to-head of ^211^At- and ^125^I-labeled farletuzumab yielded similar results. Remaining uptake at various times after the incubation is shown in Fig. 1B. The figure also shows results from the kinetic modeling (solid lines) show model results when applying equal amounts of two sets of binding parameters (one set using kon = 5.06 × 105 M^−1^ s^−1^ and koff = 5.61 × 10–3 s^−1^ and the other set using kon = 2.25 × 105 M^−1^ s^−1^ and koff = 5.02 × 10–4 s^−1^) for the mAb binding kinetics to 600,000 antigens per cell, and ignoring possible internalization.

SPR measurements for m-MeATE-conjugated farletuzumab resulted in *k*_*on*_ = 5.06 × 10^5^ M^−1^ s^−1^ and k_off_ = 5.61 × 10^−3^ s^−1^. Results for unconjugated farletuzumab resulted in similar values, i.e. 4.66 × 10^5^ M^−1^ s^−1^ and 5.81 × 10^−3^ s^−1^, respectively (Supplement Fig. 1, and Supplemental Table 1). Ebel et al. have previously presented kinetics data from SPR measurements using another method, resulting in *k*_*on*_ = 2.25 × 10^5^ M^−1^ s^−1^ and *k*_*off*_ = 5.02 × 10^−4^ s^−1^ [9].

Results from the cell experiments were analyzed and compared with both the parameter values derived from the herein presented SPR studies and with those of Ebel et al. The results indicated that the mAb binding and release kinetics were best described by a combination of the two sets of binding parameters. A good fit to all experimental results could be observed when the binding kinetics data was set with an assumption that half the available antigens bind mAb according to the kinetics described by our SPR measurements, and half bind mAb according to kinetics derived from Ebel et al [9]. With this assumption, the saturation experiments indicated roughly 600,000 available antigens per cell.

To estimate binding and penetration into microtumors, our previously presented model [8] were used with two sets of cell binding parameters, each applied to half of the estimated 600,000 available antigens. One set using *k*_*on*_ = 5.06 × 10^5^ M^−1^ s^−1^ and *k*_*off*_ = 5.61 × 10^−3^ s^−1^ and the other set using Ebel et al.'s data, i.e. *k*_*on*_ = 2.25 × 10^5^ M^−1^ s^−1^ and *k*_*off*_ = 5.02 × 10^−4^ s^−1^ [9]. Model results are shown by the solid lines in Fig. 1. The IRF studies showed a value of (0.46 ± 0.12) for ^211^At-farletuzumab on OVCAR-3. This value was lower than observed in later studies, most likely due to the probable loss of some component of bound mAbs in the washing steps in the IRF-assay. The unspecific At-rituximab was also tested on OVCAR-3 and had a IRF lower than 0.01.

### Biodistribution

**Fig. 2:**
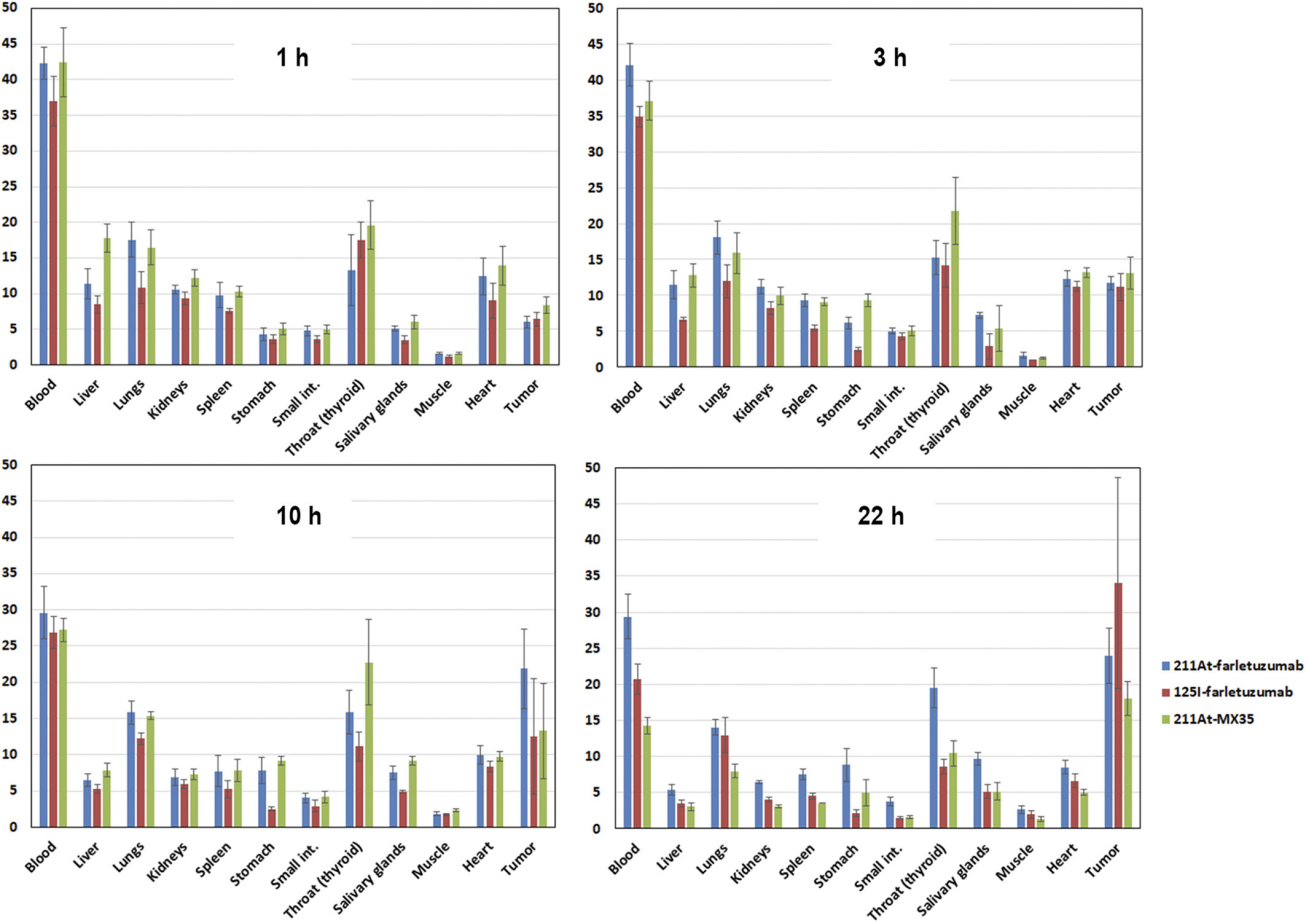
Percent injected decay-corrected activity per gram (%IA/g) in various organs of mice at 1, 3, 10 and 22 h following i.v.-injection with ^211^At-farletuzumab, ^211^At-MX35, and ^125^I-farletuzumab.

The biodistribution of ^211^At-farletuzumab was similar to that of ^125^I-farletuzumab, and also to that of ^211^At-labeled MX35 antibody (Fig. 2). For the ^211^At measurements, the higher uptakes in throat (incl. thyroid) and stomach at later time-points indicate accumulation of unlabeled, free, ^211^At. A comparison of the biodistribution of the different groups in terms of residence time is given in Suppl. Table 2.

### Alpha-camera imaging and uptake estimates

**Fig. 3:**
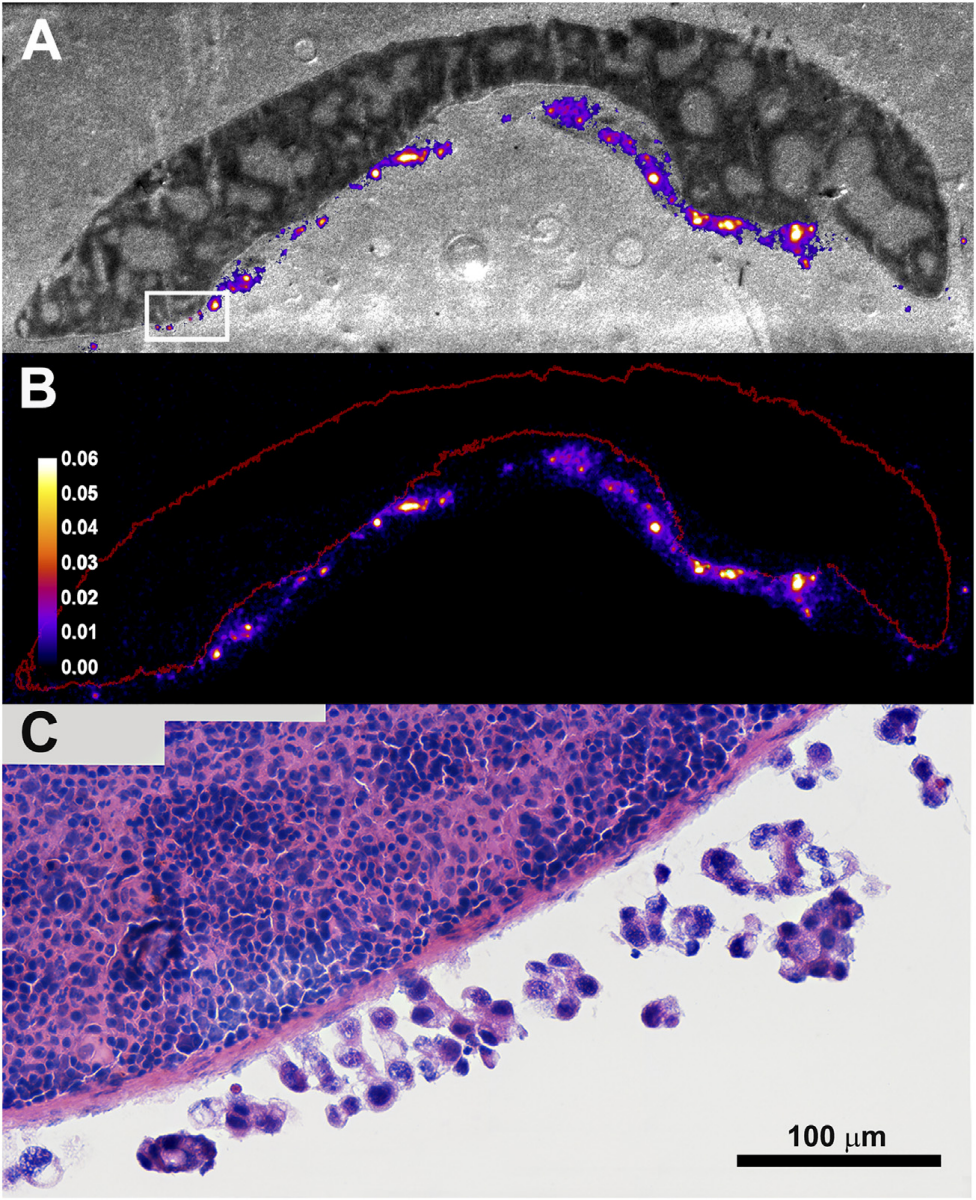
Alpha camera imaging of microtumors at 4 h after i.p. injection of ^211^At-farletuzumab (170 kBq/mL). Panel B shows an alpha image. Panel A shows the corresponding white-light photo of the imaged section, with the hot-spot areas from the alpha image superimposed. The white box in panel A is shown in panel C at 40× magnification. The ROI in red (panel B) outlines the spleen and the bright spots correspond to uptake in microtumors on the peritoneal lining of the spleen. The color-coded scale bar indicates quantified activity per cell (Bq/cell). The microtumor sizes (radius, mean ± S.D.) were 44 (±19) μm with a range from 19 to 82 μm. (For interpretation of the references to color in this figure legend, the reader is referred to the web version of this article.)

Using H&E-slides from the alpha imaging cohort of mice, the estimated microtumor size (radius, mean ± S.D.) at the day of therapy in the efficacy study was 44 (±19) μm with a range from 19 to 82 μm. Alpha imaging verified a high uptake of both ^211^At-farletuzumab and ^211^At-MX35 in microtumors, with levels up to a factor of 500 higher than for the mean for the spleen itself. Fig. 3B shows an alpha image of ^211^At-farletuzumab at 4 h after i.p. injection. Fig. 3A shows the corresponding white-light photo of the imaged section, with the hot-spot areas from the alpha image superimposed. The white box in panel A is shown in panel C at 40× magnification.

**Fig. 4:**
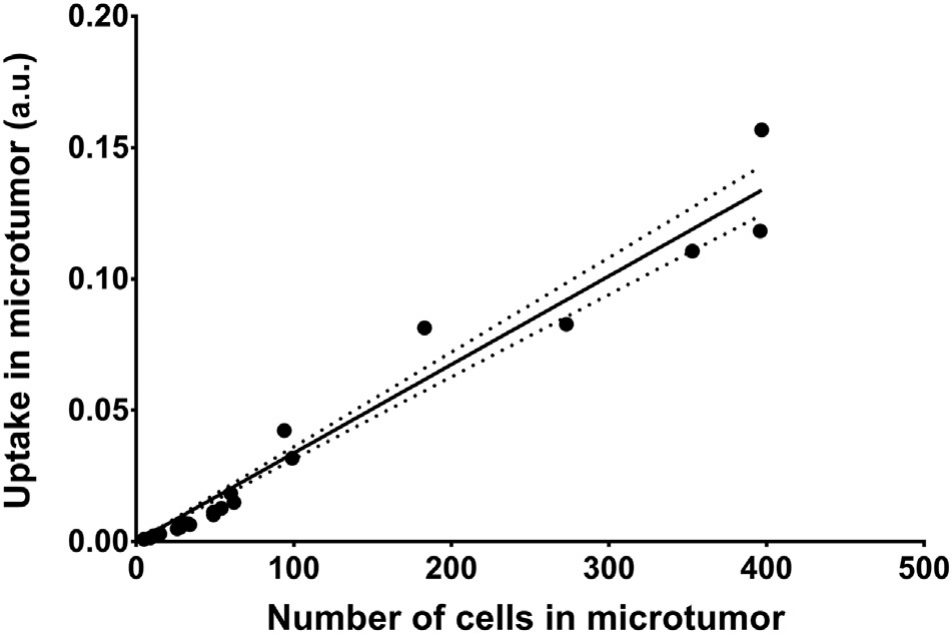
Alpha camera quantification of the total activity in each microtumor seen in Fig. 3 as a function of the number of cells. For this range of microtumor sizes (1–400 cells), the binding of ^211^At-farletuzumab per cell is relatively constant. Dotted lines indicate the 95% confidence interval for linear regression. The fitted slope was significantly different from non-zero (P-value: 0.0001) and the R-square for the goodness of fit was 0.97.

For ^211^At-farletuzumab at 4 h following i.p. injection, the activity uptake per cell was estimated for several (n = 23) microtumors. A linear relation with uptake was found for a wide range of microtumor sizes, correlating to 5–400 cells (Fig. 4), indicating that the uptake per cell is relatively constant.

### Therapeutic efficacy

A total of seven mice were euthanized before the end of the experiment, due to deteriorating health. This was evident by swollen abdomen, indicating presence of ascites. In control group C (unlabeled farletuzumab), two mice were both euthanized 99 days after therapy (d99). In control group B (unspecific ^211^At-rituximab), three mice at d120, d134, and d134. In the control group D (PBS only), two mice died atd99 and d134. All these animals presented with tumors upon dissection. Since the large majority of the mice survived the full study period any estimation of median survival was not allowed. However, when the apparent survival curves (Suppl Fig. 2) were compared a significant difference (Log-rank Mantel-Cox test) was found between ^211^At-farletuzumab and unlabeled farletuzumab (P-value = 0.04) as well as between ^211^At-farletuzumab and PBS (P-value = 0.01). The difference between ^211^At-farletuzumab and ^211^At-rituximab was not significant.

At the end of experiment, the tumor-free fraction (TFF) of the three control groups of mice (PBS, unlabeled farletuzumab, and unspecific ^211^At-rituximab) was 12%, 9% and 14%, respectively. TFF after treatment with ^211^At-farletuzumab was 91% (Table 1). The TFF following ^211^At-farletuzumab treatment was significantly different from the TFF following all control groups, i.e. ^211^At-rituximab (P < 0.0001****); unlabeled farletuzumab (P < 0.0001****); and PBS (P = 0.0001***).

**Table 1:** Tumor-free fraction of mice 154 days following treatment with specific ^211^At-farletuzumab; unspecific ^211^At-rituximab; unlabeled investigational farletuzumab; or PBS.

	Tumor-free fraction (TFF)	
Group A (n = 22); (specific) ^211^At-farletuzumab	20 of 22	91%
Group B (n = 22); (unspecific) ^211^At-rituximab	3 of 22	14%
Group C (n = 11); unlabeled investigational farletuzumab	1 of 11	9%
Group D (n = 8); PBS	1 of 8	12%

### Radiation dosimetry

**Fig. 5:**
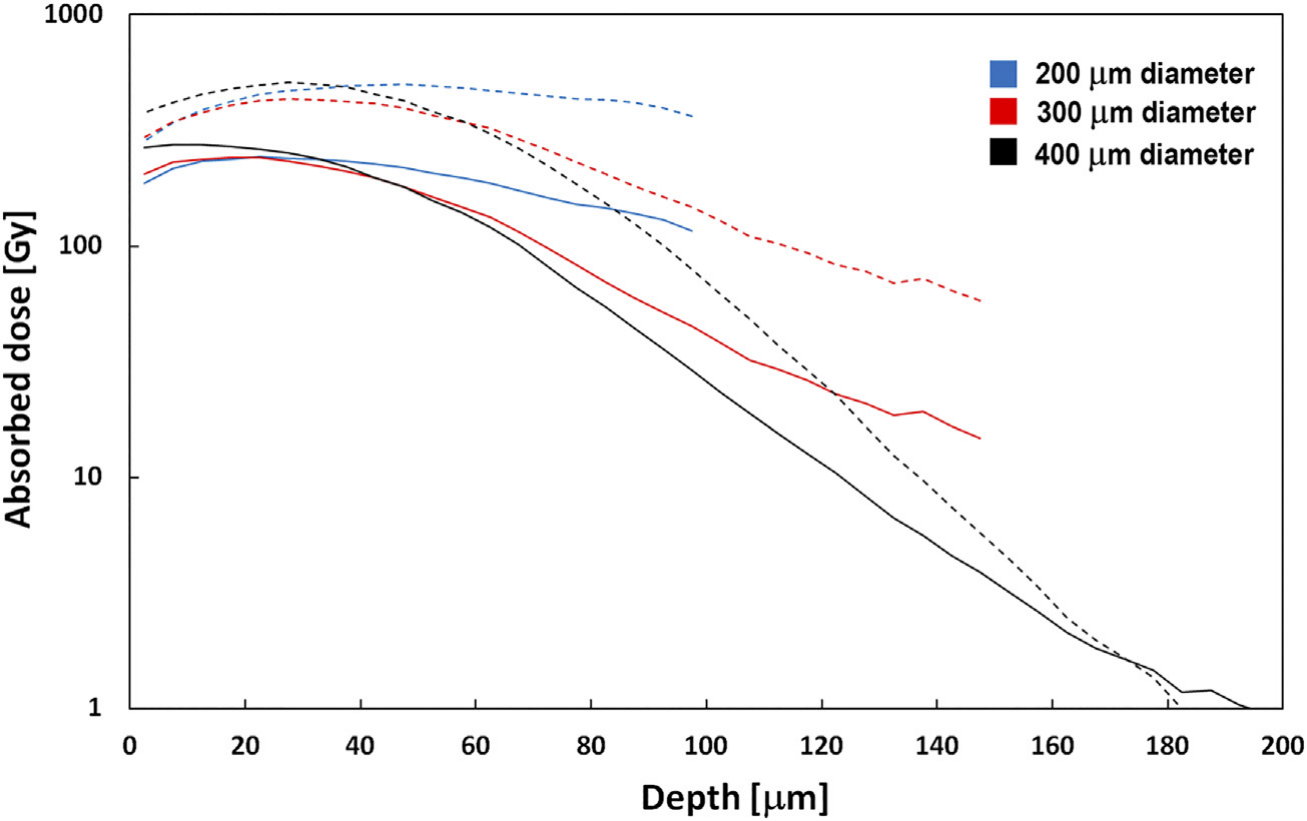
Modeled absorbed dose profiles of assumed spherical microscopic tumors of various diameters exposed to intraperitoneally administered ^211^At-farletuzumab. Dashed lines indicate modeled results for microtumors in mice exposed to amounts presented in the current work. Solid lines are model results for microtumors in patients exposed to clinically relevant amounts.

As estimated by dosimetry modeling, the radiation dose contribution from the surrounding liquid containing ^211^At-labeled mAbs (farletuzumab or rituximab), to the nucleus of a single cell within the mouse peritoneum, was 7.6 Gy. Using the binding kinetics derived within this current work, an additional 9.6 Gy would result from ^211^At-farletuzumab bound to the cell surface. The modeled dose profiles to spherical microscopic tumors of various sizes are shown in Fig. 5.

With an assumed eradicative dose at 10 Gy from alpha-particle irradiation (26), single cells and microtumors with diameters up to at least 300 μm are expected to be sterilized following treatment with ^211^At-farletuzumab. Mean absorbed doses to organs and macrotumors in the biodistribution are presented in Suppl. Table 3.

### Clinical translation

This study was designed to mimic the irradiation within the peritoneal cavity in patients treated with 200 kBq/mL intraperitoneally injected ^211^At-farletuzumab. This concentration has been established as safe with a likely therapeutic effect [4,5]. Since the retention time for antibodies within the peritoneal cavity is much shorter for mice, biokinetic modeling [7,22] show that the clinical irradiation of intraperitoneal microtumors can be simulated by injecting a higher activity concentration in mice. The therapeutic effect of 700 kBq/mL in mice is then directly translatable to a clinical situation using 200 kBq/mL (Fig. 5).

## Discussion and conclusion

The main purpose of this study was to conduct experiments and modeling that were aimed at predicting efficacy in patients. Initially, our attempts to determine the number of binding sites per OVCAR-3 cell resulted in atypical saturation curves. This made it difficult to estimate absorbed dose to microtumors in the mice, and therefore also to the patient situation. To generate results that allow an estimate of the dose to microtumors in a patient, the cell experiments were made at antibody concentrations used for clinical intraperitoneal TAT [4]. The derived binding kinetics were used, together with parameters generated from our own and Ebel et al.'s [9] SPR measurements, as input to our model that allow us to estimate microtumor dose in both mice and possible patients.

A previous study of the binding properties of farletuzumab to IGROV1 and SW620 cells concluded that it is directed against a single-class antigen [15]. Our current data, using OVCAR-3 cells, show similar binding characteristics as that presented in [15]. We found, however, that the binding to OVCAR-3 cells was best described by addition of a rapid component, making this a two-component process. The IRF ~0.5 that our experiments yield indicated a rapid component that is lost in the washings of the cells before being measured for radioactivity. The existence of a rapid component is further supported by our Biacore data. When modeling the results, a best fit was made for equal parts of the more rapid kinetics and that presented in [9]. In addition to these two components, we also observed a very slow component. This was also found in [15], where it was explained by cellular internalization. Another study found that endocytosis does not depend on the occupancy of available antigens [27], which indicates possible recycling. From our and others' [28] experience, care should thus be taken when using the method of Lindmo [21] for determining the IRF, and an assessment should be made for each case, to determine whether the method is appropriate or not.

The biodistribution study of farletuzumab showed similar behavior for both ^125^I and ^211^At as radiolabel. The organ and tumor uptakes were also similar to that of ^211^At-MX35. Another study have shown that expression of FRA on normal tissue is generally very low and mainly confined to retina, placenta, choroid plexus, and the luminal surface of kidneys and lungs [29]. These areas are not directly accessible via blood, so the distribution of ^211^At-farletuzumab to them will be delayed and any radiation damage therefore relatively low. We observed an elevated uptake in thyroid(throat) both for ^125^I and ^211^At. This is a common finding. While pre-treatment with substances such as sodium perchlorate has been investigated to mitigate this, we did not use any blocking agent in our study. We have preliminary data (unpublished) suggesting that blocking agents could infer alterations in the biodistribution causing increased toxicity in mice.

The tumor model for the therapy study was developed to simulate the clinical situation where adjuvant intraperitoneal TAT is relevant, i.e. minimal amounts of disseminated ovarian cancer within the peritoneal cavity. We have previously used this model, where a single-cell suspension is inoculated intraperitoneally, to evaluate the efficacy of other targeted alpha therapies on disseminated ovarian cancer [2,3]. Results on efficacy can be affected by antibody consumption [30,31]. In our animal model, this occurs when the amount of i.p. infused mAbs are low comparted to the number of available antigens. Since we aimed at using the same, relatively high, specific activity as for potential clinical use, we needed to ascertain that the amount of antigens on the tumor cells were not so high that the infused mAbs were “consumed” before being distributed to all available antigens. To test this, we co-injected trace amounts (10 kBq) of 125I-labeled unspecific rituximab. Blood sampling would reveal any difference in the rate with which i.p. infused specific (farletuzumab) and unspecific (rituximab) mAb distributes to the systemic circulation. If the distribution rate for the specific mAb were slower than that for unspecific mAb, this could indicate “consumption”. There was no indication of this in our experiments. Consumption is not relevant for the clinical setting where the antibody-to-antigen ratio will be much higher than in our mouse model.

By allowing two weeks from inoculation of the single-cell suspension to therapy microtumors are formed. An indirect measure of the sizes of these microtumors comes from the therapeutic efficacy of the ^211^At-bound unspecific antibody. Since roughly 10 Gy is required for microtumor eradication [8,26], our simulations suggests that tumors with diameters larger than ~200 μm were likely present at the time for therapy. Microtumors smaller than this receive absorbed doses larger than 10 Gy merely from non-specific irradiation from ^211^At decay in the peritoneal fluid.

We have previously used two months as follow-up time for the mice. In the current work we followed the mice for five months in order to facilitate a more accurate detection of remaining tumor, and thereby provide a more reliable estimate of the tumor-free fraction. Signs of toxicity were monitored at least weekly by visually inspecting and weighting the animals. In other animal models, loss of weight can indicate deteriorating health. However, in our model, such loss often occurs simultaneously with appearance of peritoneal ascites (that adds to weight) resulting in a near-zero net effect on weight. The seven mice that were euthanized before the end of experiment had all swollen abdomen, indicating presence of ascites, and presented with tumor upon dissection.

By using our previously presented modeling for the biodistribution of intraperitoneally infused antibodies in patients [7], as well as new modeling of the physiology for the mouse, we could estimate the uptake of ^211^At-farletuzumab in various sizes microtumors. These estimates were then compared with actual distribution in tumor tissue using our alpha camera [23]. Together with in-house developed software for microdosimetry [25], we could then calculate the absorbed dose to such microtumors.

In translation from mice to the patient situation, the experimental observations we have made using the intraperitoneal mouse model of ovarian cancer cohere with modeling and dosimetry calculations. This suggests that data derived in mice could be translated to what is expected in patient, when it comes to therapeutic effect on intraperitoneal microtumors. For toxicity, however, inherent differences in the pharmacological kinetics mouse versus patient make adequate translations more difficult. The main reason for this is the strong difference in passage time of a radiolabeled mAb from the intraperitoneal cavity to its systemic distribution, as illustrated in Suppl Fig. 3A. Following an i.p.-infusion of ^211^At-MX35 in a mouse the blood concentration reaches 25%IA/g already at 3 h after administration, while for a patient, using data from the previous phase I study [4], the concentration level is factor of 10^4^ lower. Correspondingly, the absorbed dose to the bone marrow following an i.p.-infusion is markedly lower in a patient than in a mouse. Accounting for their respective anticipated therapeutic administered activity (0.7 MBq/0.7 mL for mouse, 300 MBq/1500 mL for human), the dose rates to the bone marrow after infusion of ^211^At-MX35, mouse versus patient, is presented in Suppl Fig. 3B. At these activities, the dose to the bone marrow for mice can be >2000 mGy, while for patients it will be approximately 15 mGy [5].

In a clinical study on i.p. delivery of ^211^At-MX35 F(ab′)_2_ fragments, no radiation-related toxicity was seen [32]. Similar absorbed doses to healthy tissues are expected to arise from delivery of 211At-farletuzumab. Toxicity of unlabeled farletuzumab were previously studied pre-clinically [9], and the no observed adverse effect level (NOAEL) was estimated to be greater than 136.8 mg/kg, over 28 days. In contrast, the amount of farletuzumab used for i.p. therapy with ^211^At-farletuzumab on patients will be less than 1 mg in total. Toxicity of ^211^At-farletuzumab must, however, be properly studied in a clinical phase I trial. In the clinical study using the ^211^At-MX35 F(ab′)_2_ compound, we did not reach the maximal tolerated dose (MTD) [4,5]. We did not continue the dose escalation above activity concentrations of 200 MBq/L because this activity was modeled to result in microtumor doses >10 Gy [7]. These are levels anticipated to be therapeutically effective with a high probability for cure. We could clinically not demonstrate any acute toxicity while the long term effects are not known. Including long-term risks in optimizing targeted alpha therapy is not yet standard since most efforts have been placed on late-stage patients. In an adjuvant setting, however, long-terms risks must be considered as the aim is cure whereby late developing morbidity due to treatment can be of concern.

While we have attempted to evaluate the efficacy of ^211^At-farletuzumab for adjuvant intraperitoneal therapy in as clinically relevant settings as possible, some unavoidable shortcomings remains. The number of relevant antigens might vary considerable on patients' tumor cells; there might be uptake in healthy organs that does not present in mice; the stability of the compound might be compromised; and the presented models might be too simple to predict real distribution in patient. In summary, the current investigation of intraperitoneal therapy with ^211^At-farletuzumab, delivered at clinically relevant ^211^At-mAb radioactivity concentrations and specific activities, shows much promise and warrants further clinical testing.

## Funding

This study was funded by the 10.13039/501100004359Swedish Research Council (grant number 02275-2017), the 10.13039/501100002794Swedish Cancer Society (grant numbers CAN2016-752, CAN2016-761 and CAN2019-0524), the 10.13039/501100010223King Gustav V Jubilee Clinic Research Foundation (Palm-19/20) and grants from the Swedish state under the agreement between the Swedish government and the county councils, the ALF-agreement ALFGBG-435001. The investigational antibody farletuzumab was provided free of charge from Morphotek Inc., Exton, PA, USA.

## Ethical approval

All applicable international, national, and/or institutional guidelines for the care and use of animals were followed. This article does not contain any studies with human participants performed by any of the authors.

## CRediT authorship contribution statement

**Stig Palm:** Conceptualization, Methodology, Software, Validation, Formal analysis, Investigation, Writing - original draft, Writing - review & editing, Visualization, Supervision, Project administration, Funding acquisition.
**Tom Bäck:** Conceptualization, Methodology, Software, Validation, Formal analysis, Investigation, Writing - review & editing, Visualization.
**Emma Aneheim:** Methodology, Formal analysis, Investigation, Writing - review & editing, Visualization.
**Andreas Hallqvist:** Writing - review & editing.
**Ragnar Hultborn:** Conceptualization, Writing - review & editing, Supervision.
**Lars Jacobsson:** Conceptualization, Methodology, Software, Validation, Formal analysis, Writing - review & editing, Visualization, Supervision.
**Holger Jensen:** Resources, Writing - review & editing.
**Sture Lindegren:** Methodology, Investigation, Writing - review & editing, Funding acquisition.
**Per Albertsson:** Conceptualization, Methodology, Writing - review & editing, Visualization, Supervision, Project administration, Funding acquisition.

## Declaration of competing interest

The authors declare the following financial interests/personal relationships which may be considered as potential competing interests: Stig Palm, Tom Bäck, Lars Jacobsson, Sture Lindegren and Per Albertsson declare ownership in the Swedish commercial company Aprit Biotech AB. The data in this manuscript was derived prior to the declared ownership.

## Appendix A Supplementary data

Supplementary material Image 1
