# Supplementary material for: Evaluation of therapeutic efficacy of 211At-labeled farletuzumab in an intraperitoneal mouse model of disseminated ovarian cancer
Source: Transl Oncol. 2020 Sep 25;14(1):100873. doi: 10.1016/j.tranon.2020.100873 (PMC7522120; doi:10.1016/j.tranon.2020.100873)
Supplement: Supplementary file 1 — Supplementary material [file mmc1.docx]

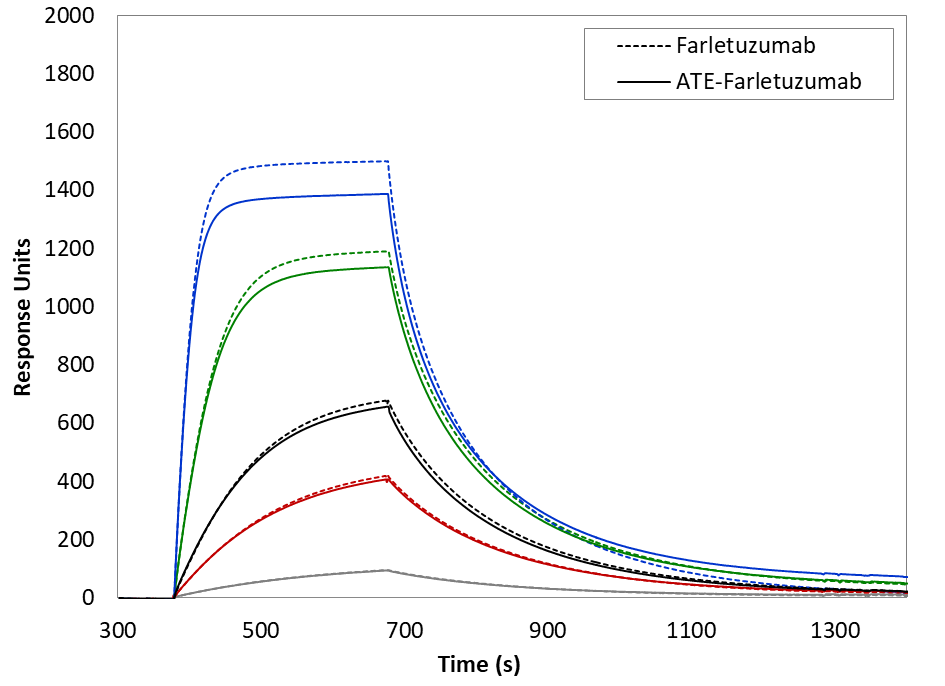


***Supplemental Fig 1.*** *SPR measurements (Biacore 2000) of farletuzumab and m-MeATE-farletuzumab immobilized on a Protein A chip, measuring binding of Folate alpha (concentrations 1 (grey), 5 (red) 10 (black, average of three), 30 (green) and 80 (blue) nM) with complete chip regeneration after each concentration.*

|  | *k_on_* (M^-1^s^-1^ ) | *k_off_* (s^-1^) | *K_D_ (nM)* |
| --- | --- | --- | --- |
| Murine LK26(9) | 4.65×10^5^ | 3.25×10^-3^ | 7.0 |
| Humanized LK26(9) | 8.19×10^4^ | 1.70×10^-2^ | 208 |
| MORAb-003(9) | 2.25×10^5^ | 5.02×10^-4^ | 2.2 |
| Unconjugated farletuzumab | 4.66×10^5^ | 5.81×10^-3^ | 12.5 |
| m-MeATE-conjugated farletuzumab | 5.06×10^5^ | 5.61×10^-3^ | 11.1 |

***Supplemental Table 1.*** *Comparison of binding kinetic parameters*

|  | **Mean organ weight** | **AUC^*^**  (Bq ∙ s ∙ 10^6^) | | | **Residence Time^**^**  (min) | | |
| --- | --- | --- | --- | --- | --- | --- | --- |
|  | (mg) | ^211^At-farletuzumab | ^211^At-MX35 | ^25^I-farletuzumab | ^211^At-farletuzumab | ^211^At-MX35 | ^125^I-farletuzumab |
| Blood | 359 | 2975 | 2646 | 5185 | 70,8 | 63,0 | 123,4 |
| Salivary glands | 136 | 224 | 216 | 512 | 5,3 | 5,1 | 12,2 |
| Throat | 30 | 108 | 145 | 307 | 2,6 | 3,4 | 7,3 |
| Heart | 96 | 243 | 247 | 488 | 5,8 | 5,9 | 11,6 |
| Lungs | 131 | 504 | 456 | 957 | 12,0 | 10,8 | 22,8 |
| Stomach | 157 | 242 | 299 | 658 | 5,8 | 7,1 | 15,7 |
| Small Intestine | 247 | 253 | 251 | 495 | 6,0 | 6,0 | 11,8 |
| Liver | 557 | 1143 | 1379 | 2479 | 27,2 | 32,8 | 59,0 |
| Spleen | 115 | 227 | 218 | 435 | 5,4 | 5,2 | 10,4 |
| Kidneys | 234 | 483 | 465 | 887 | 11,5 | 11,1 | 21,1 |
| Tumor | 68 | 246 | 199 | 517 | 5,8 | 4,7 | 12,3 |
| Muscle | 116 | 48 | 46 | 112 | 1,1 | 1,1 | 2,7 |

***Supplemental Table 2.*** *Mean organ weights, area-under-curve (AUC; given as cumulated activity) and residence times per organ for the different radiolabeled antibodies in the biodistribution study. The data represents an injected activity of 0.7 MBq.*

**AUC per organ was calculated from the uptake data (%IA/g) over time using the trapezoidal rule, accounting for each organs respective mean weight.*

***Residence time was derived by dividing the organ AUC by the injected activity.*


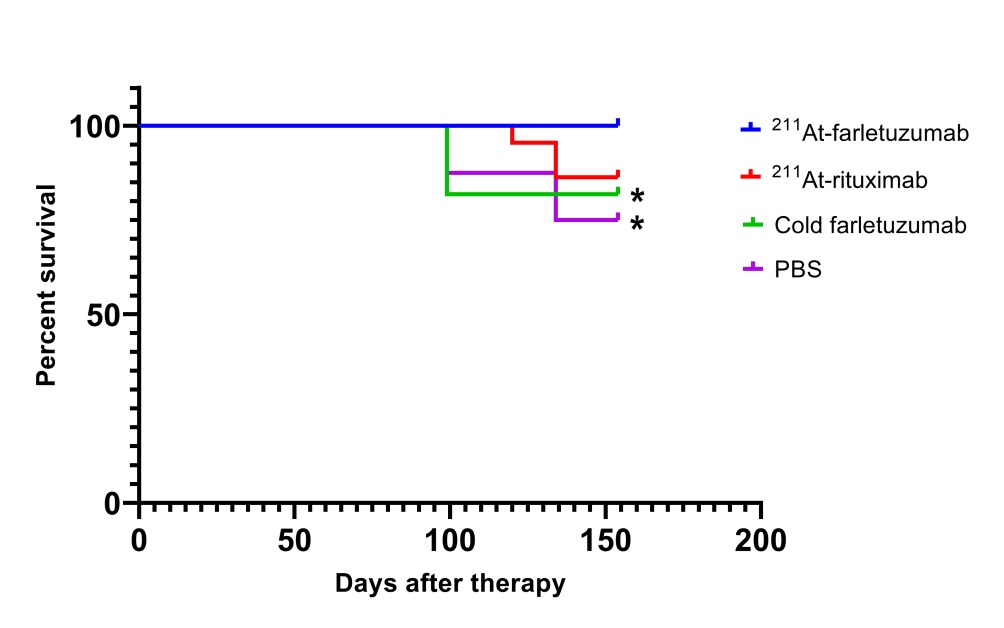


***Supplemental Fig 2.*** *Plot of the apparent survival for the different treated in the therapy study. * indicate groups for which the survival curves were significantly different from that of the 211At-farletuzumab-group.*

| Mean absorbed dose (Gy/0.7 MBq) | |
| --- | --- |
| Blood | 9,0 |
| Bone marrow* | 3,1 |
| Salivary glands | 1,8 |
| Throat** | 3,9 |
| Lungs | 4,2 |
| Stomach | 1,7 |
| Small Intestine | 1,1 |
| Liver | 2,2 |
| Spleen | 2,1 |
| Kidneys | 2,2 |
| Tumor | 3,9 |

***Supplemental Table 3.*** *Mean absorbed doses to organs and macrotumors in the biodistribution study.*

**Dose to the bone marrow was calculated from the blood concentration assuming a Bone marrow-to-Blood-ratio of 0.3.*

***The thyroid was not removed from the throat*


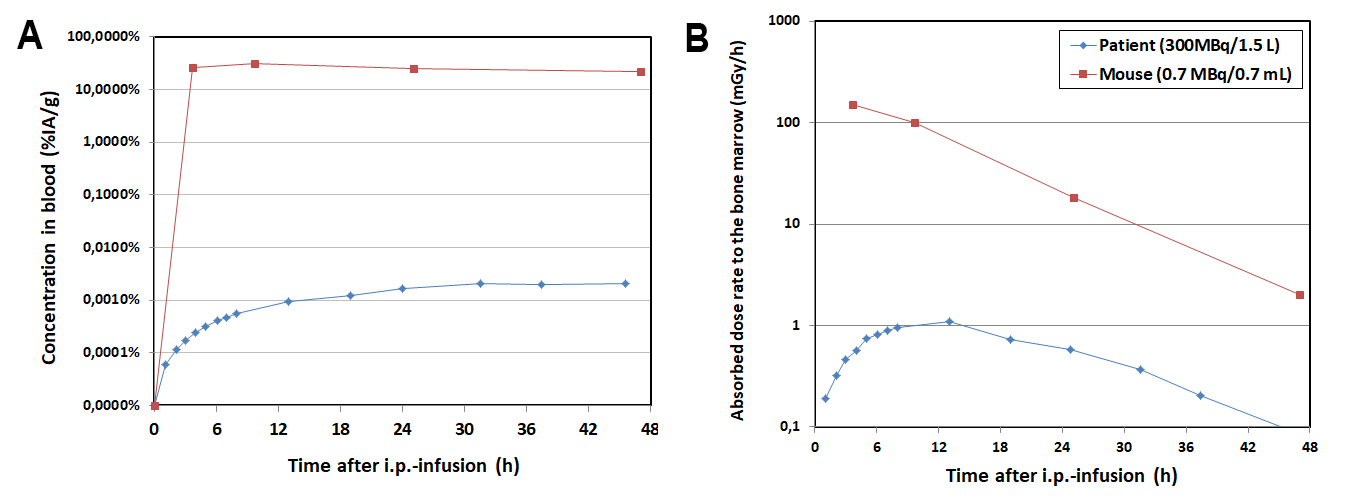


***Supplemental Fig 3.*** *(A) Comparison of the increase in blood concentration of ^211^At-MX35 following an i.p.-infusion in a mouse versus in a patient. (B) Comparison of the corresponding differences in absorbed dose rates to the bone marrow in a mouse versus in a patient. The data represents their respective anticipated therapeutic administered activity (0.7 MBq/0.7 mL for mouse, 300 MBq/1500mL for human).The patient data used was from (4).*
